# Supplementary material for: Region matters: Mapping the contours of undernourishment among children in Odisha, India
Source: PLoS One. 2022 Jun 10;17(6):e0268600. doi: 10.1371/journal.pone.0268600 (PMC9187075; doi:10.1371/journal.pone.0268600)
Supplement: S1 File — (PDF) [file pone.0268600.s001.pdf]

Fig 1- 22: Odisha Block-wise Univariate and Bivariate LISA results

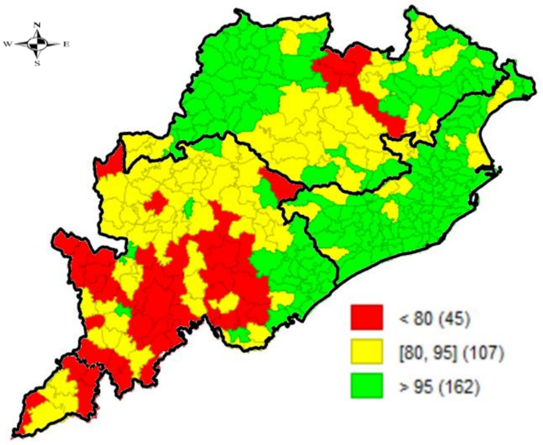

Fig 1 Prevalence of Women receiving a postnatal check within 48 hours of delivery, block-wise, CCM II, Odisha

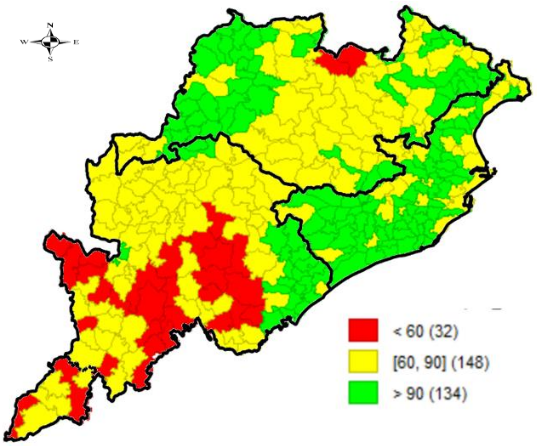

Fig 2 Prevalence of Institutional deliveries, block-wise, Odisha

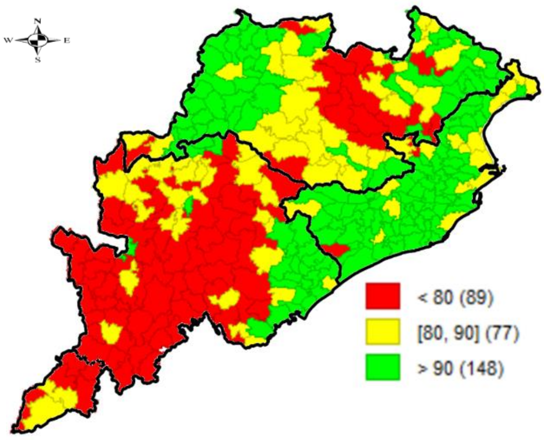

Fig 3 Prevalence of skilled births, block-wise, Odisha

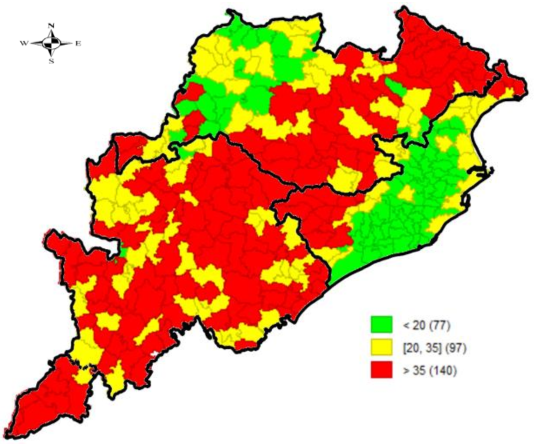

Fig 4 Prevalence of women's first pregnancy before 19 years of age, block-wise, Odisha

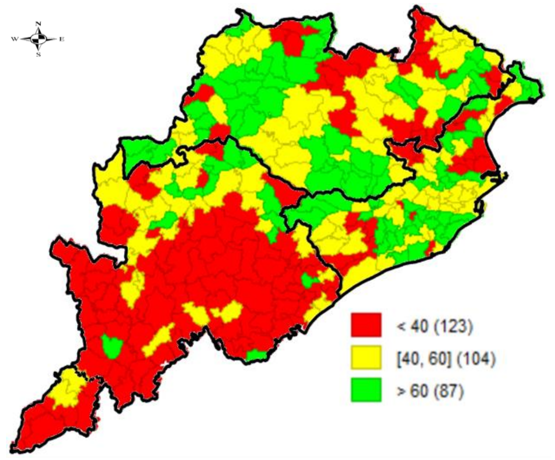

Fig 5 Prevalence of women having more than 4 ANC check-ups, block-wise, Odisha

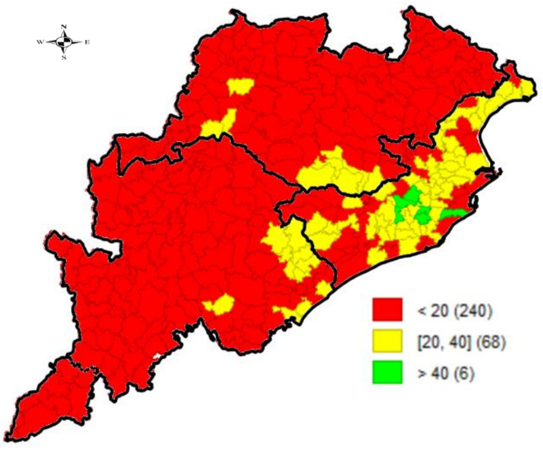

Fig 6 Prevalence of households with improved sanitation, block-wise, CCM II, Odisha

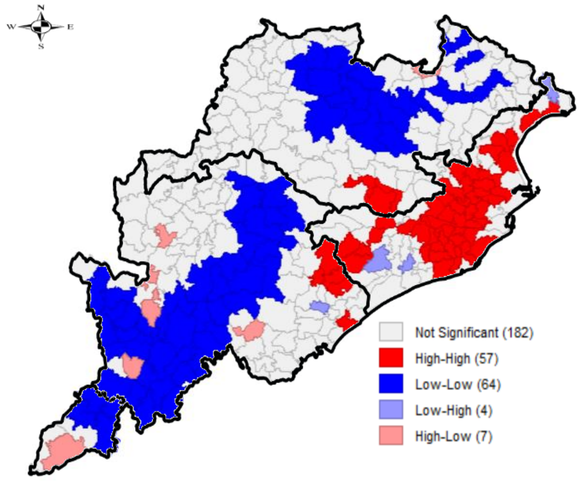

Fig 7 Univariate LISA cluster map for improved sanitation, block-wise, Odisha CCM II

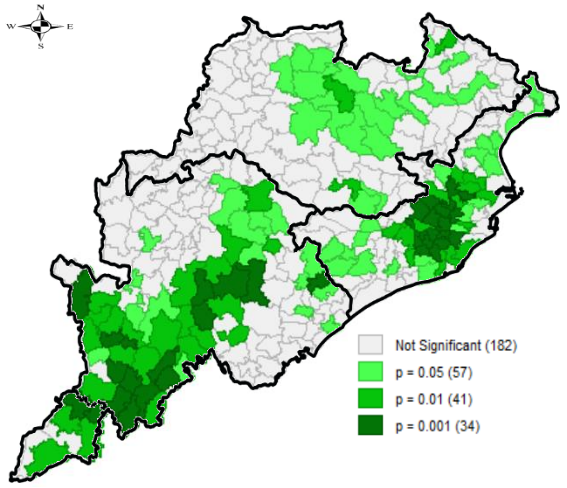

Fig 8 Univariate LISA significance map for improved sanitation, block-wise, Odisha CCM II

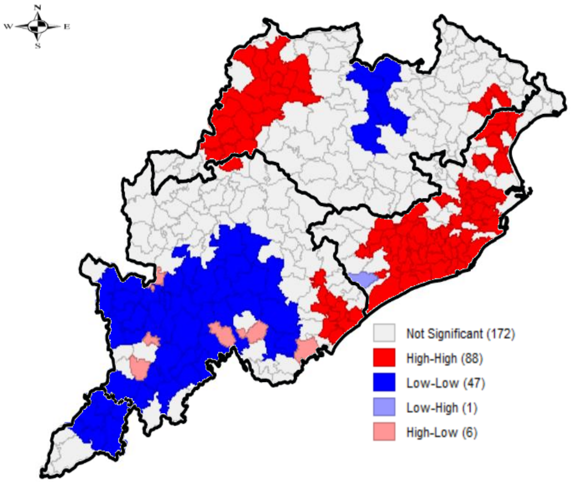

Fig 9 Univariate LISA cluster map for postnatal check-ups, block-wise, Odisha CCM II

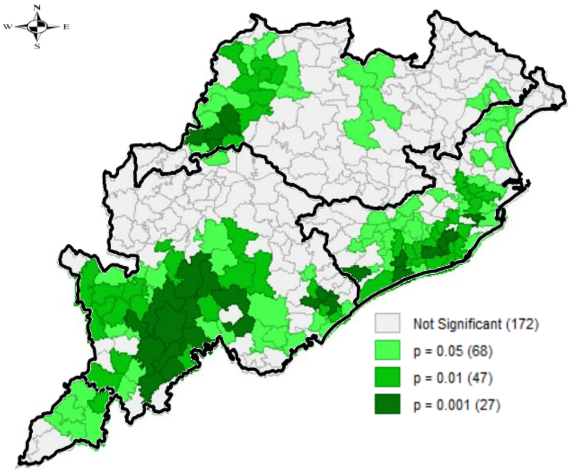

Fig 10 Univariate LISA significance map for postnatal check-ups, block-wise, Odisha CCM II

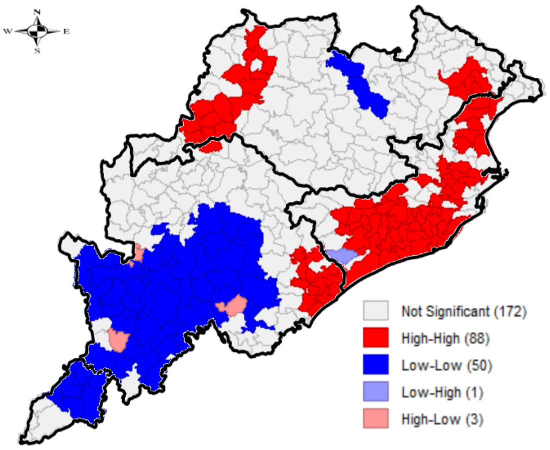

Fig 11 Univariate LISA cluster map for institutional deliveries, block-wise, Odisha CCM II

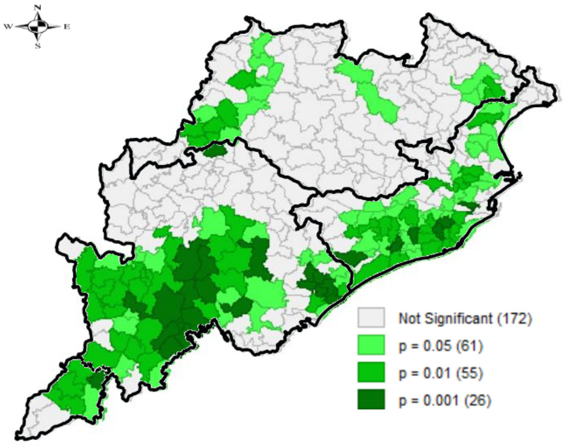

Fig 12 Univariate LISA significance map for institutional deliveries, block-wise, Odisha CCM II

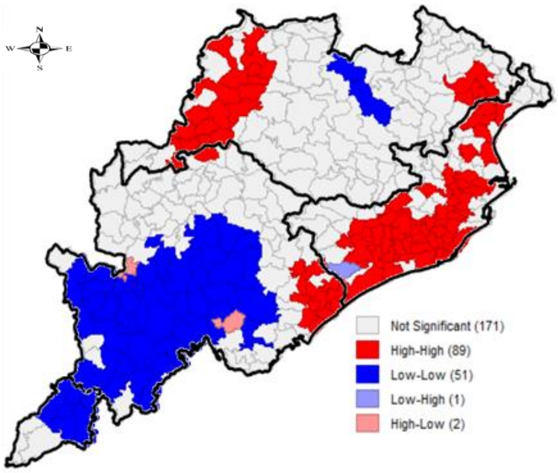

Fig 13 Univariate LISA cluster map for skilled births, block-wise, Odisha CCM II

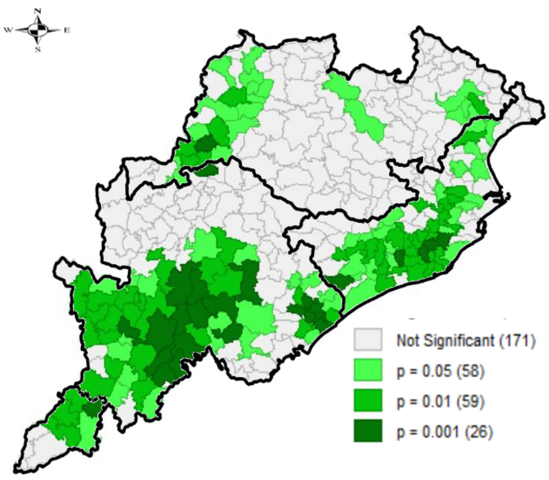

Fig 14 Univariate LISA significance map for skilled births, block-wise, Odisha CCM II

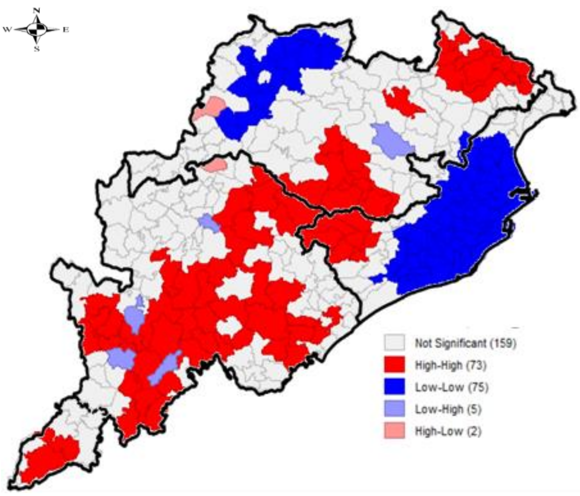

Fig 15 Univariate LISA cluster map for first pregnancy before the age of 19, block-wise, Odisha CCM II

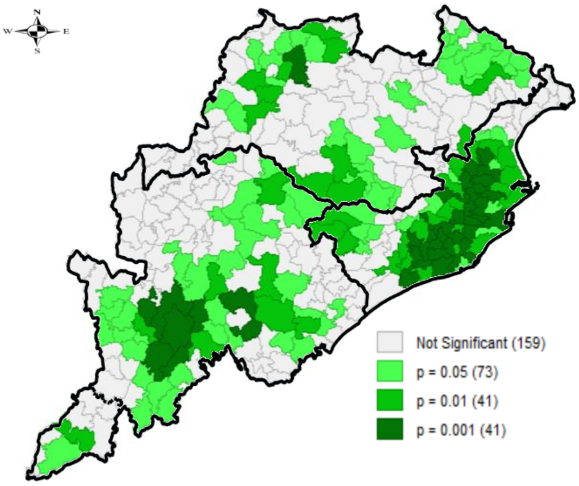

Fig 16 Univariate LISA significance map for first pregnancy before the age of 19, block-wise, Odisha CCM II

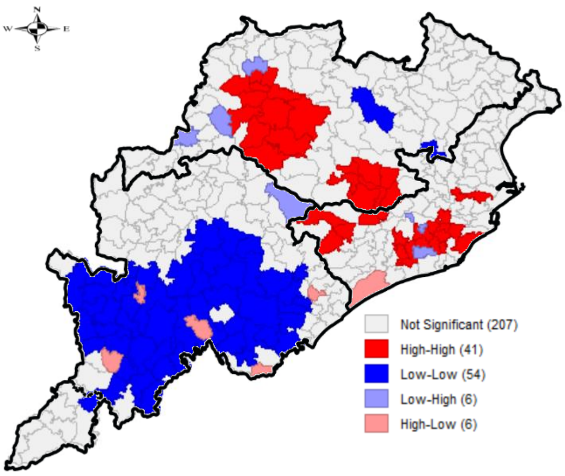

Fig 17 Univariate LISA cluster map for more than 4 ANC check-ups, block-wise, Odisha CCM II

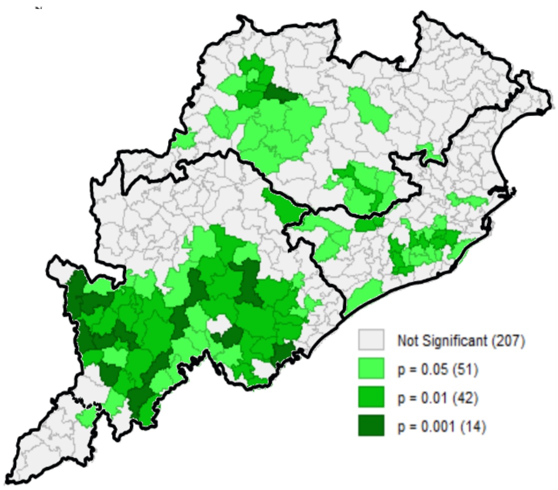

Fig 18 Univariate LISA significance map for more than 4 ANC check-ups, block-wise, Odisha CCM II

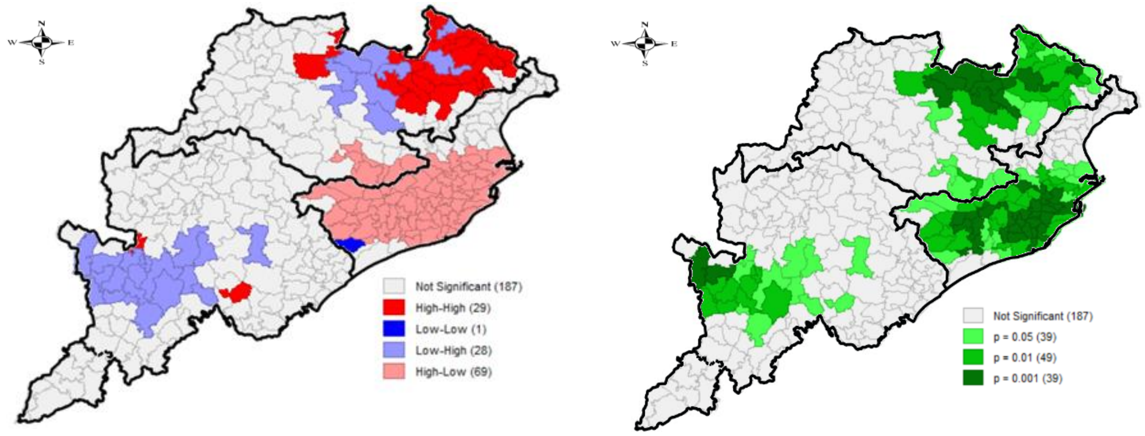

Fig 19 Bivariate LISA cluster and significance map of Underweight vs Institutional delivery, Odisha CCM II

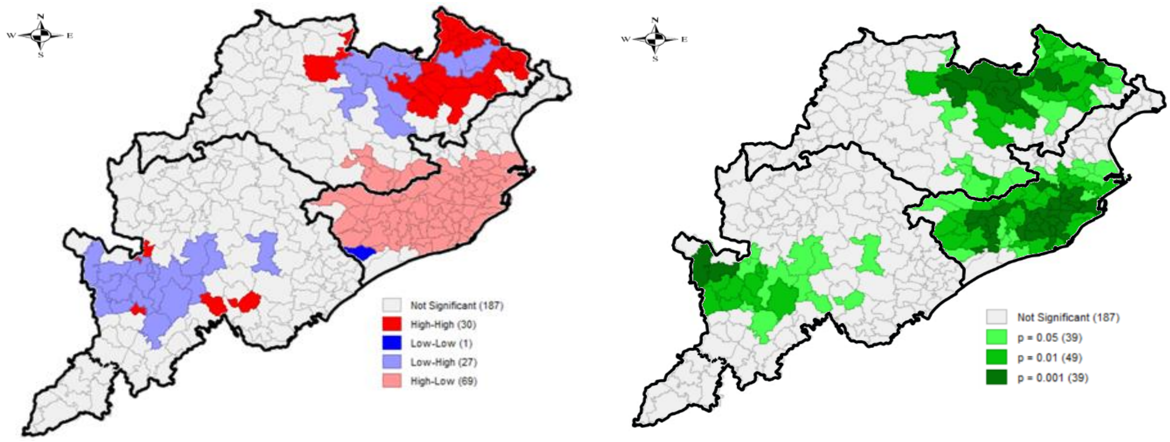

Fig 20 Bivariate LISA cluster and significance map of Underweight vs Women receiving a postnatal check within 48 hours of delivery, Odisha CCM II

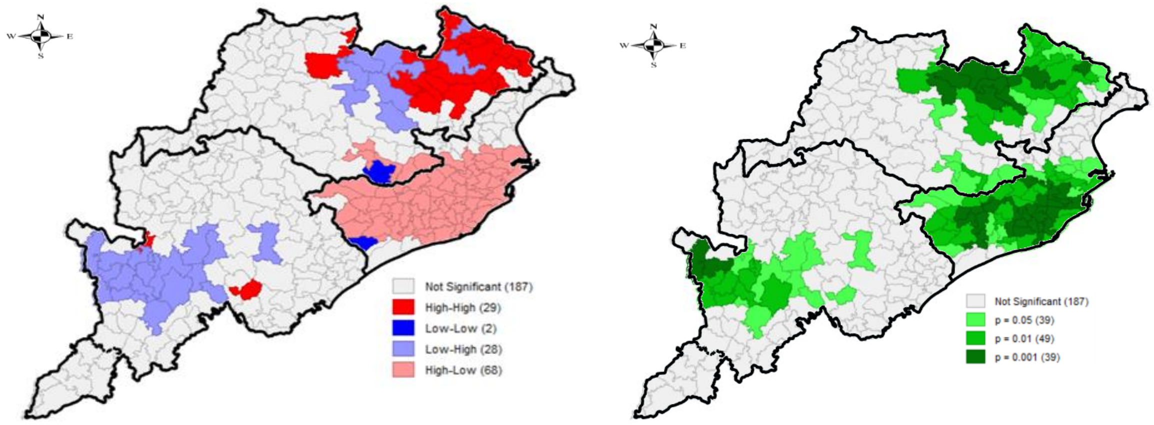

Fig 21 Bivariate LISA cluster and significance map of Underweight vs Skilled birth attendance, Odisha CCM II

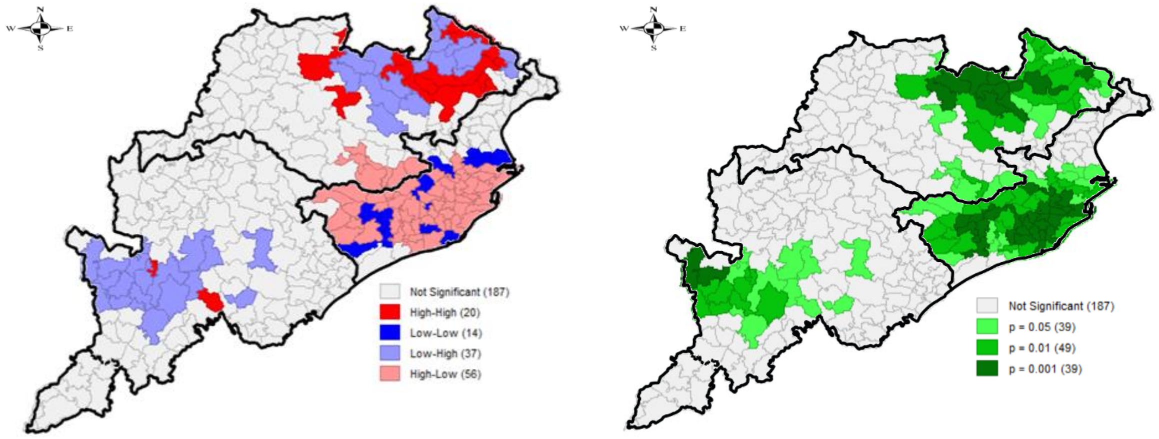

Fig 22 Bivariate LISA cluster and significance map of Underweight vs >4 ANC, Odisha CCM II
