# Supplementary material for: Region matters: Mapping the contours of undernourishment among children in Odisha, India
Source: PLoS One. 2022 Jun 10;17(6):e0268600. doi: 10.1371/journal.pone.0268600 (PMC9187075; doi:10.1371/journal.pone.0268600)
Supplement: S2 File — (PDF) [file pone.0268600.s002.pdf]

Differences across NSS regions of Odisha: NFHS-4 Unit level analysis

Table 1, depicts the population distribution according to the socio-economic indicators, as per the NSS natural regions of Odisha. From the NFHS4 data, the total children sample as per the regions was noted as 2,671 for the coastal region, 4,005 for southern and 3,052 for northern. The prevalence of children underweight was observed the highest in the northern region at 40.6%, followed by the southern region having 39.5% prevalence, and the lowest prevalence in the coastal region at 23.6%.

Table 1 Profile of the study population, as per the NSSO regions, Odisha, NFHS-4

Malnutrition indicators & correlates	Coastal		Southern		Northern	
	%	N	%	N	%	N
**Children underweight**						
Underweight	23.6	632	39.5	1,583	40.6	1,239
**Age of the child in months**						
0-5	7.4	199	8.5	341	7.4	227
6-11	9.3	250	10.4	416	9.9	302
12-15	5.8	155	6.4	255	6.5	198
16-18	5	133	5.9	235	4.7	143
19-23	8.3	222	8.3	333	8.8	269
24-35	20.8	557	20.6	824	22.4	683
36-47	21.3	569	20.2	808	20.8	633
48-59	22	587	19.8	792	19.5	596
**Type of place of residence**						
urban	17.2	460	11.4	458	15.9	486
rural	82.8	2,211	88.6	3,547	84.1	2,566
**Sex of child**						
male	52	1,388	51.4	2,058	50.8	1,549
female	48	1,283	48.6	1,947	49.2	1,503
**Social Group**						
SC	24.8	663	24	961	16.7	509
ST	7.1	189	34.2	1,372	42.8	1,307
OBC	35.7	954	31.7	1,271	30	917
others	32.4	865	10	402	10.4	319
**Wealth index**						
poorest	25.5	681	47.9	1,920	44.4	1,356
poorer	28.3	756	25.1	1,005	25	762
middle	26.3	703	14.8	594	15.7	479
richer	14.8	396	8.1	324	9.8	299
richest	5.1	136	4	162	5.1	155
**Mother's highest educational level**						
no education	11.8	315	44.4	1,779	27.4	836
primary	13.9	372	16.1	645	13.2	403
secondary	67.8	1,812	36.1	1,447	53.2	1,624
higher	6.4	172	3.4	135	6.2	189
**Mother's current age**						
below 20	1.8	48	3.3	132	3.2	98
20-24	27.1	725	32	1,280	28.5	870
25 & above	71.1	1,899	64.7	2,593	68.3	2,084

Malnutrition indicators & correlates	Coastal		Southern		Northern	
	%	N	%	N	%	N
**Birth order number**						
1	49.8	1,330	37.1	1,487	43.6	1,332
2	34.8	929	31.1	1,244	33.3	1,018
3	9.8	263	16.7	671	12.9	393
4 & above	5.6	149	15.1	603	10.2	310
**Preceding birth interval (months)**						
<12	50.4	1,347	37.9	1,519	44.7	1,365
12-23	6.9	183	8.3	334	7.4	225
24-35	10.5	279	17.8	715	15.3	466
36&above	32.2	861	35.9	1,438	32.6	996
**Type of toilet facility**						
Improved	38.9	1,040	19.3	774	27.2	831
Not Improved	61.1	1,631	80.7	3,231	72.8	2,221
**Nutritional Status of Mother**						
BMI>=18.5	78.5	2,095	68	2,719	69.7	2,125
BMI<18.5	21.5	575	32	1,281	30.3	923
**No. of ANC visits**						
No ANC	7.4	170	5.7	184	6.8	175
<= 3ANC	34.1	780	31.9	1,019	26.2	671
>= 4ANC	58.5	1,337	62.4	1,994	67	1,716
**Place of Delivery**						
Institutional	93.1	2,487	78.7	3,151	86.6	2,642
Non-Institutional	6.9	184	21.3	854	13.4	410
Total		2,671		4,005		3,052

Bivariate analysis in the form of cross-tabulation and Pearson's chi-square test to check the association, were carried out to examine the prevalence of undernutrition based on the socio-economic indicators and maternal background characteristics, by the three NSSO natural regions separately, and are presented in Table 2.

Table 2 Prevalence and association of children underweight; by background, child and maternal characteristics according to the Odisha NSSO regions, NFHS-4 2015-16

Background, Child and Maternal Characteristics	Coastal	Southern	Northern
**Age of the child in months**			
0-5	22.6	31.9	32.4
6-11	19.1	30.4	34.5
12-15	25.3	36.1	35.7
16-18	18.8	38.2	46.6
19-23	19.5	40.9	43.7
24-35	22.1	41.1	42
36-47	28.3	40.5	41.1
48-59	25.1	45.8	43.4
	Pearson $\chi2(7)$ = 17.4214	Pearson $\chi2(7)$ = 47.7073	Pearson $\chi2(7)$ = 16.7240
	Pr = 0.015	Pr = 0.000	Pr = 0.019
**type of place of residence**			
urban	19.4	25	35.9
rural	24.5	41.4	41.5
	Pearson $\chi2(1)$ = 6.9559	Pearson $\chi2(1)$ = 33.0455	Pearson $\chi2(1)$ = 11.1578
	Pr = 0.008	Pr = 0.000	Pr = 0.001
**wealth index**			
poorest	34.7	46.2	49.2
poorer	28.7	42.4	41.9

Background, Child and Maternal Characteristics	Coastal	Southern	Northern
middle	17.1	29.7	28.7
richer	12.7	22	29.2
richest	6.3	13.8	17.7
	Pearson χ2(4) = 120.3123	Pearson χ2(4) = 145.1539	Pearson χ2(4) = 125.0932
	Pr = 0.000	Pr = 0.000	Pr = 0.000
**sex of child**			
male	23.1	39.7	42.7
female	24.2	39.4	38.4
	Pearson χ2(1) = 0.7554	Pearson χ2(1) = 1.4037	Pearson χ2(1) = 4.6426
	Pr = 0.385	Pr = 0.236	Pr = 0.031
**Social Group**			
SC	30.6	35.4	40.1
ST	42.1	47.6	50.9
OBC	20.9	37.9	32.2
others	17.3	26.8	23.3
	Pearson χ2(3) = 78.3401	Pearson χ2(3) = 35.0060	Pearson χ2(3) = 99.7243
	Pr = 0.000	Pr = 0.000	Pr = 0.000
**Type of toilet facility**			
Improved	16.1	27.6	31
Not Improved	28.5	42.4	44.2
	Pearson χ2(1) = 50.9288	Pearson χ2(1) = 45.1504	Pearson χ2(1) = 43.1518
	Pr = 0.000	Pr = 0.000	Pr = 0.000
**birth order number**			
1	20.5	38.9	35.5
2	23.4	32.6	40.5
3	31.9	45.2	48.2
4 & above	38.6	49	53.2
	Pearson χ2(3) = 36.3116	Pearson χ2(3) = 46.8794	Pearson χ2(3) = 43.3555
	Pr = 0.000	Pr = 0.000	Pr = 0.000
**preceding birth interval (months)**			
<12	20.6	39	35.6
12-23	27.8	40.2	52.5
24-35	32	42.3	47.6
36&above	24.8	38.5	41.5
	Pearson χ2(3) = 19.6087	Pearson χ2(3) = 2.8862	Pearson χ2(3) = 40.9602
	Pr = 0.000	Pr = 0.410	Pr = 0.000
**Mother's highest educational level**			
no education	41.3	46.5	54.9
primary	31.4	42.7	45
secondary	20.6	31	34.5
higher	6.1	24	20.1
	Pearson χ2(3) = 116.0276	Pearson χ2(3) = 76.0035	Pearson χ2(3) = 117.0943
	Pr = 0.000	Pr = 0.000	Pr = 0.000
**Mother's current age**			
below 20	34.2	41.3	37
20-24	24.8	35.3	38.8
25 & above	22.9	41.5	41.5
	Pearson χ2(2) = 4.5246	Pearson χ2(2) = 9.6121	Pearson χ2(2) = 2.4020
	Pr = 0.104	Pr = 0.008	Pr = 0.301
**Nutritional Status of Mother**			
BMI>=18.5	19.9	33.4	34.9
BMI<18.5	37.2	52.6	53.6
	Pearson χ2(1) = 76.0082	Pearson χ2(1) = 133.9338	Pearson χ2(1) = 100.8641
	Pr = 0.000	Pr = 0.000	Pr = 0.000
**No. of ANC visits**			
No ANC	27.2	37.3	53.5
<= 3ANC	23.4	39.4	40.5
>= 4ANC	22.8	39.2	39.2
	Pearson χ2(2) = 2.0196	Pearson χ2(2) = 0.3862	Pearson χ2(2) = 7.6149
	Pr = 0.364	Pr = 0.824	Pr = 0.022
**Place of Delivery**			
Institutional	22.9	37.3	38.3
Non-Institutional	33.9	47.6	55.4
	Pearson χ2(1) = 13.0644	Pearson χ2(1) = 26.1219	Pearson χ2(1) = 29.7175
	Pr = 0.000	Pr = 0.000	Pr = 0.000

Background, Child and Maternal Characteristics	Coastal	Southern	Northern
**Total**	23.6	39.5	40.6

* P<0.10; ** P<0.05 & *** P<0.01 level of significance
