# Supplementary figures and images for: Region matters: Mapping the contours of undernourishment among children in Odisha, India
Source: PLoS One. 2022 Jun 10;17(6):e0268600. doi: 10.1371/journal.pone.0268600 (PMC9187075; doi:10.1371/journal.pone.0268600)

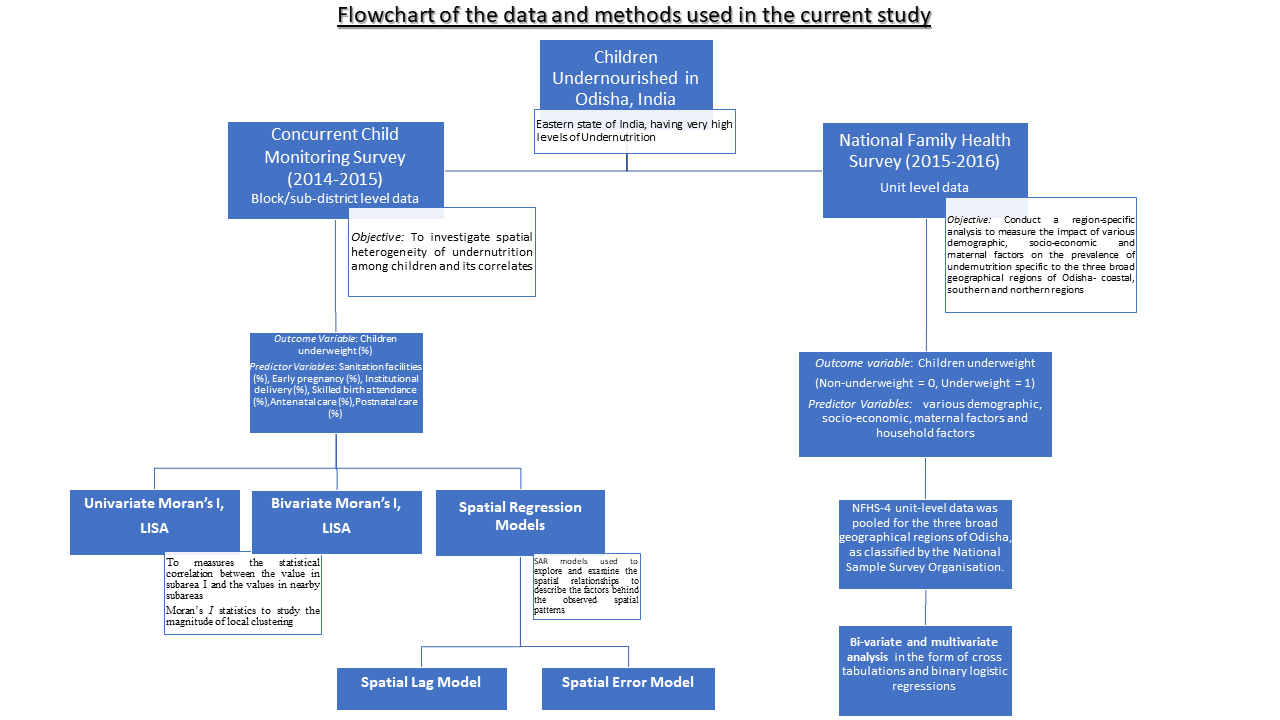

Supplement: S1 Fig — (TIF) [file pone.0268600.s003.tif]
